# ^1^H Nuclear Magnetic Resonance of Lodgepole Pine Wood Chips Affected by the Mountain Pine Beetle

**DOI:** 10.3390/ma4010131

**Published:** 2010-12-31

**Authors:** Tara M. Todoruk, Ian D. Hartley, Roshanak Teymoori, Jianzhen Liang, Hartwig Peemoeller

**Affiliations:** 1 Physics Department, University of Northern British Columbia, 3333 University Way, Prince George, British Columbia, Canada; E-Mail: todorukt@unbc.ca; 2 Ecosystem Science and Management Program, University of Northern British Columbia, 3333 University Way, Prince George, British Columbia, Canada; 3 Department of Chemistry, University of Alberta, 11227 Saskatchewan Drive, Edmonton, Alberta, Canada; E-Mail: teymoori@ualberta.ca; 4 Department of Physics and Astronomy, University of Waterloo, 200 University Avenue West, Waterloo, Ontario, Canada; E-Mail: peemoell@uwaterloo.ca

**Keywords:** free induction decay (FID), lodgepole pine, moisture content, mountain pine beetle, nuclear magnetic resonance (NMR), spin-spin relaxation time

## Abstract

In this study, wood-water interactions of mountain pine beetle affected lodgepole pine were found to vary with time since death. Based on an analysis of magnetization components and spin-spin relaxation times from ${}^{1}$H NMR, it was determined that the mountain pine beetle attack does not affect the crystalline structure of the wood. Both the amorphous structure and the water components vary with time since death, which could be due to the fungi present after a mountain pine beetle attack, as well as the fact that wood from the grey-stage of attack cycles seasonally through adsorption and desorption in the stand.

## 1. Introduction

Wood-water interactions have been studied extensively for many years [1,2,3]. Because wood is a hygroscopic material, exposure to different atmospheric conditions, such as temperature or humidity, results in an equilibrium moisture content (EMC). The EMC is dependent on other factors such as previous exposure history, mechanical stress, extractives present, and species. Furthermore, the EMC of wood conditioned at a given relative humidity (RH) is always higher during the desorption phase than in the adsorption phase, with the initial desorption being highest [2,3].

There are numerous methods used for measuring the moisture content (MC) and the dry-basis (equation 1) in wood, such as the gravimetric method, distillation method, Karl Fischer titration method, electrical moisture meters, nuclear energy, and proton nuclear magnetic resonance (${}^{1}$H-NMR) [2,3]. ${}^{1}$H-NMR is unique in the sense that information can be extracted about both the wood and water components. Using ${}^{1}$H-NMR, it is possible to determine the amount of liquid per amount of solid material [4]. NMR provides insight into the interactions between wood and water, as well as information about the molecular motions and state of the water in relation to the macroscopic materials [5]. Furthermore, ${}^{1}$H-NMR provides insight into the ratio of “bound” and “free” water within wood [6], as well as the ratio of crystalline and amorphous solids, as these can be distinguished in a free induction decay (FID) [7]. Previously, the characterization of crystalline and amorphous material was performed using high-field NMR, although more recently, low-field NMR has proven to be suitable [8,9,10,11]. More specifically, from the FID, the spin-spin relaxation times (T${}_{2}$) can be extracted. As T${}_{2}$s can be sensitive to the mobilities of protons, the protons present in different structures, such as water, crystalline solids, and amorphous solids, would have different T${}_{2}$s [12]. As the T${}_{2}$s vary, it is possible to extract information about each separate component within the wood. In wood conditioned to EMCs below the fibre saturation point (FSP), it would be expected that only “bound” water exists, and therefore only one water component should be seen below this point [2].

In recent years, British Columbia (BC) has experienced a mountain pine beetle (*Dentroctonus ponderosae* Dougl. Ex Loud.) epidemic, which has affected millions of cubic meters of standing timber of lodgepole pine (*Pinus contorta* var. *latifolia*) trees. After a tree has experienced an attack, it exhibits stages of dying, classified as “time since death” (TSD) stages. The TSD stages characterize the stage of attack as red (MPB-R) and grey (MPB-G). In the red stage, the tree shows initial signs of dying, such as needles being red in colour, and takes 2–4 years. In the grey stage, the tree has lost its needles, appearing grey in colour, and has been standing dead for 5 or more years [13,14].

Mountain pine beetle (MPB) affected wood has been examined by many researchers to determine the effect of the blue stain fungi produced from the attack on its physical properties. Although many of the physical properties are not affected by the beetle attack [14], previous research indicates that sorption properties are affected by the attack, specifically due to the fungi present in the wood [15]. ${}^{1}$H-NMR has been used for early detection of enzymatic fungi attack on paper [16], although the mechanism of attack of blue-stain fungi is not enzymatic and does not degrade the cell wall [17].

In the wood products industry, the effect of MC is important in the manufacturing process and the final product performance. The objective of this study was to determine the wood-water interactions within MPB affected wood using both a T${}_{2}$ analysis, as well as a component fraction analysis. The component fraction analysis was performed to determine the signal contribution of each component within the wood, while the T${}_{2}$ analysis was performed in order to provide information about states (liquid, solid, crystalline, amorphous) of the molecular systems containing the ${}^{1}$H nuclei as well as the molecular mobility.

## 2. Results and Discussion

${}^{1}$H-NMR experiments were performed on lodgepole pine of different stages of mountain pine beetle attack, conditioned in both adsorption and initial desorption to various moisture contents. The FID was analyzed and fitted to Equation 2, which includes terms for both crystalline and amorphous solid components, and a water component. As the samples were all conditioned below the FSP, only one water component was expected. From the fit, a component analysis and a T${}_{2}$ analysis were performed.

### 2.1. Magnetization Component Analysis

**Figure 1:**
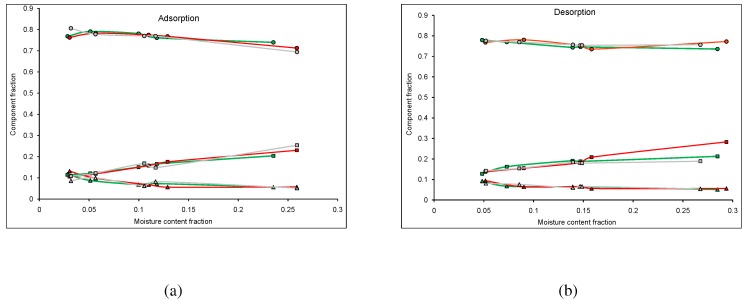
Fractional component analysis for mountain pine beetle affected lodgepole pine conditioned in initial desorption, where the circles correspond to the NMR signal component fitted to the sinc function, the triangles correspond to that fitted to the gaussian function, and the squares correspond to that fitted to the exponential function. The colours correspond to the stage of attack; unaffected (green), red stage (red), and grey stage (grey). The standard error in the points was negligible compared to the size of the symbols used to represent the data.

A fractional component analysis was performed on three different TSDs, MPB-R, MPB-G, and un-attacked lodgepole pine (LP), conditioned in both adsorption and initial desorption. The three components analyzed, Equation 2, include a crystalline solid component, an amorphous solid component, and a water component, with adsorption shown in Figure 1a and initial desorption shown in Figure 1b.

For both adsorption and desorption, the crystalline solid component (sinc function) contributes the highest fraction of the FID, and the amorphous solid component (gaussian function) contributes the lowest fraction of the FID. The water component (exponential function) contributes approximately the same fraction of the FID as the amorphous solid component at low MCs but increases substantially with increased MC.

For adsorption, wood from all TSDs have similar fractions for all three components. This suggests that the MPB attack does not strongly change the relative contribution from the wood and water components in adsorption (Figure 1a). There is more variation in the water components at high MCs, although this variation is small. Any variation of the water component could be due to the presence of blue-stain fungi within the MPB wood [15].

For desorption, wood from all TSDs appear to have similar component fractions for all three components (Figure 1b). Wood from all TSDs have approximately the same signal contribution for the solid components, suggesting that the MPB attack does not impact this contribution. However, at high MCs, the MPB-G has a lower water contribution, while the MPB-R has a higher contribution. The increased contribution in the MPB-R wood could be attributed to the increased presence of fungi in the red stage. As fungi exhibits strong sorption properties, one would expect a higher water component when more fungi is present. This supports previous work suggesting that sorption isotherms increase when blue-stain fungi is present in the wood [15]. Furthermore, the lower contribution in the MPB-G wood could be attributed to the seasonal adsorption/desorption cycling occurring in the stand.

Comparing the component fractions for adsorption and desorption, the signal contribution of the solid components for all three TSDs remain relatively constant, with only the contribution of the water component showing any substantial change. The contribution of the water component for the MPB-G wood for both adsorption and desorption are similar, which could be due to the adsorption/desorption cycling occurring in the MPB-G wood in the stand, where the water contribution for the MPB-R and LP wood are higher during desorption and lower during adsorption. This difference could be due to the hysteresis effect, as the desorption for both the LP and MPB-R was truly initial, as no cycling occurred.

### 2.2. Spin-Spin Relaxation Time Analysis

**Figure 2:**
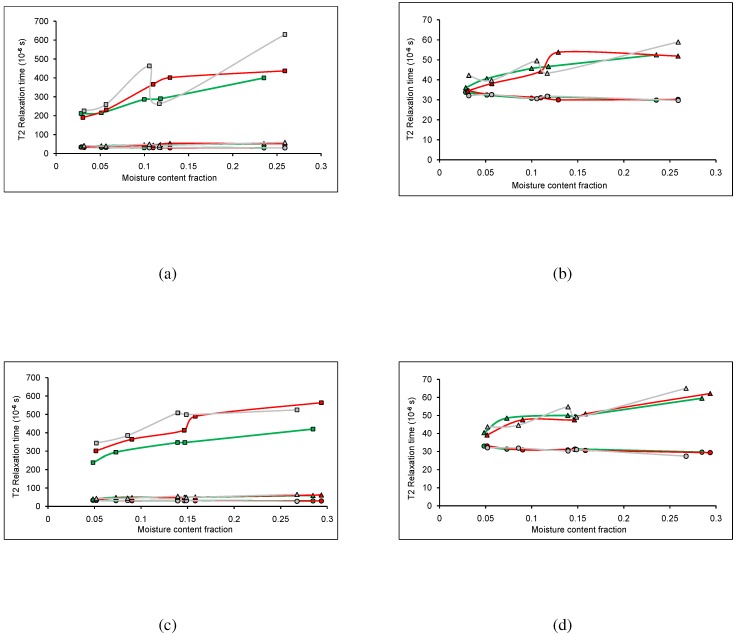
Spin-spin relaxation time (T${}_{2}$) analysis for mountain pine beetle affected lodgepole pine, where the circles correspond to the relaxation time from the gaussian broadened sinc function, the triangles correspond to the relaxation time from the gaussian function, and the squares correspond to the relaxation time from the exponential function. The colours correspond to the stage of attack; unaffected (green), red stage (red), and grey stage (grey). **(a)** T${}_{2}$ values for wood conditioned in adsorption; **(b)** Magnification of scale from (a); **(c)** T${}_{2}$ values for wood conditioned in initial desorption; **(d)** Magnification of the scale from (c). The standard error on the points was negligible compared to the size of the symbols used to represent the data.

A spin-spin relaxation time analysis was performed on wood chips from three TSDs conditioned in both adsorption and initial desorption. The T${}_{2}$s from crystalline solid, amorphous solid, and water were analyzed, with adsorption shown in Figure 2a and Figure 2b and initial desorption shown in Figure 2c and Figure 2d.

For both adsorption and desorption, the longest T${}_{2}$s were attributed to water (exponential function), and the shortest T${}_{2}$s were attributed to crystalline solid (sinc function). The T${}_{2}$s attributed to the water and amorphous solid increased with increased moisture content, which would be expected as water interacts with the amorphous structure within wood and not the crystalline structure.

For adsorption (Figure 2a) and desorption (Figure 2c), more variation in the T${}_{2}$ values was observed than in the fractional components. In both sorption cases, the T${}_{2}$ of water in MPB-R and MPB-G had a considerably longer relaxation time than in LP. For adsorption the water in MPB-G exhibited a higher relaxation time than in MPB-R (except at ∼12% MC) (Figure 2a), whereas for desorption the MPB-G had a longer T${}_{2}$ than MPB-R below ∼18% MC and shorter T${}_{2}$ above ∼18% (Figure 2c). As the water in MPB-R and MPB-G had a higher relaxation time on average than the LP, the interaction of water within the wood itself was dependent on TSD. Previously, it was seen that the adsorption isotherms for MPB affected wood were higher than for LP, possibly due to the presence of blue-stain fungi, as the fungi has a higher water affinity for water than wood [15]. The difference in T${}_{2}$s for the water could be due to the presence of the fungi, as well. The desorption isotherms for MPB affected wood were higher for MPB-R than for LP, and higher for MPB-G than for LP under ∼12%, possibly due to the presence of blue-stain fungi, and partially as the MPB-G experiences seasonal adsorption/desorption cycling within the stand and the desorption was truly not initial [15]. This could account for the differences observed in the water T${}_{2}$s.

As shown in Figure 2b,d, the T${}_{2}$s for the solid material are similar for all three stages of attack. For both sorption cases, the T${}_{2}$s for the crystalline solid remained consistent for all TSDs, suggesting that the MPB attack does not affect the crystalline structure in the wood. There was more variation seen in the T${}_{2}$s for the amorphous solid. For adsorption, the MPB-R exhibits longer T${}_{2}$s over the moisture content range of 12% to 20%, with MPB-G exhibiting longer T${}_{2}$s at higher moisture contents (Figure 2b). For desorption, the MPB-G exhibited longer T${}_{2}$s at high moisture contents, with LP exhibiting much lower T${}_{2}$s at higher moisture contents (Figure 2d). This suggests that the MPB attack does affect the amorphous structure in the wood.

When comparing the relaxation times between adsorption and desorption, it can be seen that the T${}_{2}$s for the crystalline structures are consistent for all TSDs in both adsorption and desorption. This means that the sorption method, as well as the MPB attack does not change the crystalline structure within the wood. Both the amorphous structure and the water T${}_{2}$s do vary more for adsorption and desorption, as well as with the MPB attack. This suggests that these components within the wood are more sensitive to the sorption method, as well as the MPB attack. Note that in adsorption, anomalies occur at ∼12% MC and that in desorption they occur at ∼14% MC. These anomalies are typically seen at these MCs and while theories exist explaining these anomalies, the cause of them is not understood entirely.

## 3. Experimental Section

Lodgepole pine logs from the central interior of BC were obtained based on the TSD-stages, namely the red (MPB-R) and grey (MPB-G), as well as a log of non-attacked lodgepole pine (LP) to use as a control sample. The logs were chipped whole, and no difference was made between sapwood and hardwood. The average size of the wood chips during sorption was 2.77 mm (standard error = 0.20 mm) in thickness, 17.52 mm (standard error = 1.15 mm) perpendicular to the grain, and 21.41 mm (standard error = 0.46 mm) along the grain. As these samples were too large to perform spectroscopy on, they were cut down immediately prior to performing the spectroscopy in order to preserve the EMCs, and included a variety of wood chips.

The wood chips were conditioned in a steady humid environment to specific moisture contents using desiccators filled with various salt solutions. The salts used were: lithium chloride (LiCl·H${}_{2}$O, RH = 15%); calcium chloride (CaCl${}_{2}$, RH = 32.3%); sodium dichromate (Na${}_{2}$Cr${}_{2}$O${}_{7}$·2H${}_{2}$O,RH = 52%); sodium nitrite (NaNO${}_{2}$, RH = 66%); and sodium sulfate anhydrous (Na${}_{2}$SO${}_{4}\cdot$10H${}_{2}$O, RH = 93%). In each desiccator, a stir bar was added to the salt solution to ensure a stable humidity.

For desorption, the samples were conditioned in the desiccators with no prior conditioning. For adsorption, the samples were oven-dried in a convection oven for 24 hours at 103 ± 2 ${}^{\circ }$C, then placed into a desiccator containing anhydrous calcium sulfate (Drierite${}^{®}$) to allow the samples to cool to room temperature without any gain in moisture, before being placed into a desiccator containing a salt solution.

These samples were monitored regularly to determine the EMC. To do this, each sample was briefly removed from the desiccator and weighed as quickly as possible on a digital scale (±0.01 g), as to maintain the RH in each desiccator. The EMC was taken as the point when the mass remained constant for a minimum of 48 hours, and the samples were left in the desiccators for a minimum of 48 hours after the EMC was reached to ensure stability. The conditioning was done at a constant room temperature of 22 ± 2 ${}^{\circ }$C.

The dry-basis MC fraction, *m*, is calculated using:

(1) $$m=\left(\frac{w-w_{od}}{w_{od}}\right)$$

where *w* is the weight of the sample at each specific humidity condition and $w_{od}$ was the weight of the sample at the oven-dry condition.

${}^{1}$H-NMR spectroscopy was performed using a spectrometer operating at 26 MHz with a receiver recovery time of 7 ms, and a bandwidth of 1 MHz to collect the FID. The FID was obtained through the application of a single 90${}^{\circ }$ radio frequency pulse of 3.5 *μ*s duration. The ${}^{1}$H-NMR signal was obtained using 1,000 point accumulation in order to maximize the precision. The sample temperature during spectroscopy measurements was maintained at 23.1 ± 0.2 ${}^{\circ }$C.

**Figure 3:**
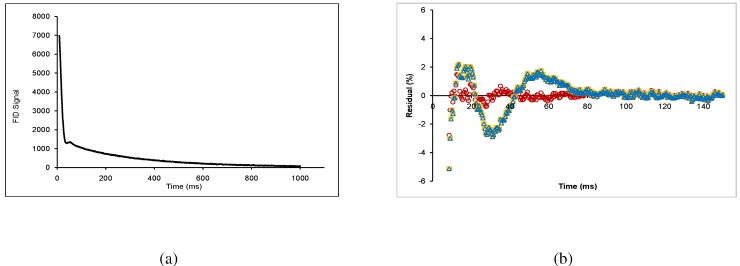
**(a)** Free-induction decay of lodgepole pine wood chips conditioned at RH = 73%; **(b)** Typical residuals plot, where the red circles correspond to Equation 2, the orange squares correspond to a combination of gaussian broadened sinc function with an exponential, and the blue triangles correspond to a gaussian function with an exponential.

The FID (Figure 3a) was fitted to an equation composed of a gaussian-broadened sinc function, a gaussian function, and an exponential function:

(2) $$y=w_{s}*\left(sin\left(x\right)\right)/\left(x\right)*exp\left(-x^{2}/T_{2s}^{2}\right)+w_{g}*exp\left(-{\left(x/T_{2g}\right)}^{2}\right)+w_{e}*exp\left(-x/T_{2e}\right),$$

where $w_{s}$, $w_{g}$, and $w_{e}$ are the weights of the sinc, gaussian, and exponential components respectively, and $T_{2s}$, $T_{2g}$, and $T_{2e}$ are the relaxation times of the sinc, gaussian, and exponential components respectively. The sinc function describes the beat pattern, which occurs in inorganic crystals and glasses, as well as carbohydrate glasses [5]. Knowing that the chemical makeup of wood is complex, this could correspond to crystalline and ordered amorphous components. The term “ordered amorphous” suggests that the short-range organization of molecules can never be completely random [5], and therefore can exhibit effects of rigid molecules. The gaussian function also corresponds to a solid component, presumably a non-ordered amorphous component of the sample [11,18,19]. The exponential function corresponds to liquid in the sample [20]. Combinations of these terms have been used previously, such as a gaussian-broadened sinc function and an exponential [21,22], or a gaussian function and an exponential [23,24] for different materials, however it was found that the sum of the all three terms provided a closer fit (see residuals given in Figure 3b). The data sets were fit using the non-linear regression procedure in SigmaStat (Version 3.11) to a tolerance of $1.0\times 10^{-7}$ using 1000 iterations to determine the variables. A component fraction analysis was performed similarly to Maus *et al*. [10]. In this study, the values plotted in Figure 1 were the fractional contributions of each component to the overall signal ($w_{s}/\left(w_{s}+w_{g}+w_{e}\right)$ for the sinc component, $w_{g}/\left(w_{s}+w_{g}+w_{e}\right)$ for the gaussian component, and $w_{e}/\left(w_{s}+w_{g}+w_{e}\right)$ for the exponential component).

## 4. Conclusions

Wood-water interactions of mountain pine beetle affected lodgepole pine are found to vary with time since death. The contribution to the signal was found to be constant for the solid components for both adsorption and desorption, and the contribution to the signal for the water components was found to vary for desorption. The spin-spin relaxation times were constant for the crystalline solid, and similar for the amorphous solid in both adsorption and desorption, but showed large variation for the water components. This suggested that the crystalline structure was not affected by the mountain pine beetle attack, while the amorphous structure and the water components were. This could be due to the fungi present after a mountain pine beetle attack, as well as the fact that wood from the grey-stage of attack cycles through adsorption and desorption in the stand.
